# Neurotrophic and Neurotoxic Effects of Aβ42 and Its Oligomers on Neuronal Survival: Revealed by Their Opposite Influence on the Potency of Extracellular BDNF

**DOI:** 10.3390/ijms26104501

**Published:** 2025-05-08

**Authors:** He Li, Changxin Zheng, Kai Wen, Tianyu Zhang, Yingjiu Zhang

**Affiliations:** Key Laboratory for Molecular Enzymology and Engineering of the Ministry of Education, School of Life Sciences, Jilin University, Changchun 130012, China; lhe20@mails.jlu.edu.cn (H.L.); zhengcx22@mails.jlu.edu.cn (C.Z.); wenkai23@mails.jlu.edu.cn (K.W.); zty18@mails.jlu.edu.cn (T.Z.)

**Keywords:** amyloid-β protein (Aβ42), brain-derived neurotrophic factor (BDNF), catechins (CAs), branched oligosaccharides (BOs), tyrosine kinase receptor B (TrkB), p75, Alzheimer’s disease (AD)

## Abstract

Brain-derived neurotrophic factor (BDNF) is critical for neuronal survival. Amyloid-β monomers (Aβ42M) and oligomers (Aβ42O) have trophic and toxic effects on neuronal survival, respectively. Branched oligosaccharides (BOs) and catechins (CAs) can specifically bind to Aβ42M/Aβ42O, influencing both effects. However, whether and how Aβ42M/Aβ42O influences BDNF remains unknown. This study investigated the interaction between Aβ42M/Aβ42O and BDNF, the effects of Aβ42M and Aβ42O on BDNF binding to the TrkB/p75 receptor and their impact on BDNF-supported cell survival, and the roles of BOs and CAs in these processes. BDNF exhibited stronger binding affinity for Aβ42M and Aβ42O than BOs/CAs. Aβ42M increased neuronal viability by synergistically enhancing BDNF binding to TrkB and p75, whereas Aβ42O decreased neuronal viability by inactivating/consuming BDNF, thereby reducing its binding to these receptors. BDNF-Aβ42O binding appeared to mutually neutralize/counteract each other’s biological effects; therefore, increasing BDNF levels might reduce Aβ42O’s neurotoxicity. By competitively targeting Aβ42M/Aβ42O rather than BDNF or its receptors, BOs and CAs enhanced these effects. These findings suggest that Aβ42M’s neurotrophicity was directly linked to its synergistic enhancement of BDNF activity, whereas Aβ42O’s neurotoxicity was primarily due to its inactivation or consumption of BDNF. This study provided valuable insights for developing BOs/CAs-based neuroprotective therapeutics or nanomaterials against AD.

## 1. Introduction

Alzheimer’s disease (AD) is a complex neurodegenerative disorder characterized by the loss of hippocampal neurons and the presence of various types of amyloid-β protein 1–42 (Aβ42) aggregates and amyloid plaques, primarily in the hippocampus. Since the C-terminal fragment of the Aβ42 molecule is hydrophobic [1,2], Aβ42 monomers (Aβ42M) tend to self-assemble into aggregates in the absence of molecular chaperone-like substances, gradually forming physiologically harmful oligomers (Aβ42O), protofibrils (Aβ42P), fibrils (Aβ42F), and plaques, even though they function like neurotrophins for neuronal survival and development [3,4]. These Aβ42 aggregates, particularly Aβ42O, are the primary cause of neuronal loss in AD [5], disrupting synapses and causing neuronal death both in vitro and in vivo. Therefore, the main medications should prevent Aβ42 from aggregating and depositing or stop Aβ42 aggregates from causing injury and reducing cell viability [6].

Aβ42M has beneficial effects on various properties of neuronal cells, such as supporting cell survival [4,7], motility, and adhesion [8,9]. In contrast, Aβ42 aggregates adversely affect multiple aspects of neuronal function and directly disrupt a range of membrane receptor-mediated pathways [10]. Although the physiological functions of these Aβ42 species are primarily manifested in their effects (whether positive or negative) on neuronal cell viability or survival, the key mechanisms underlying these effects remain poorly understood, significantly hindering the development of effective prevention and treatment strategies for AD.

Neurotrophins (NTs), called growth factors, are essential for synaptic plasticity and mediate neuron survival and development in the peripheral and central nervous systems (CNS). Brain-derived neurotrophic factor (BDNF), nerve growth factor (NGF), NT3, and NT4/5 are the four main NTs in the brain. In addition to preserving neuronal viability and function, NTs—particularly BDNF—contribute to the repair of injured cells in adults [11,12,13]. Thus, injured but viable CNS neurons can be repaired with strong support from neurotrophic factors. The impairment and death of neurons occur when effective BDNF levels are insufficient or when its binding to TrkB (tyrosine kinase receptor B) and p75 receptors is blocked, which is a major cause of the onset and progression of neurodegenerative diseases [13]. Therefore, NTs—particularly BDNF—may be a key or primary target for AD prevention and treatment [14].

Clarifying whether Aβ42 or its aggregates specifically interact with BDNF and how these interactions influence the cytological effects of BDNF or its correlation with neuronal survival is crucial, as both Aβ42 and BDNF significantly impact neuronal viability. We recently found that branched oligosaccharides (BOs), particularly isomaltotriose (IG3) and bianntennary N-linked core pentasaccharide (BCP), enhance neuronal survival by functioning as molecular chaperone-like substances for Aβ42M or antagonistic-binding partners for Aβ42O [15]. Additionally, catechins (CAs), especially catechin (CA) and epigallocatechin gallate (EGCG), have been reported to bind to Aβ42 and attenuate the cytotoxicity associated with Aβ42 aggregates [16,17,18]. CAs are a class of tea polyphenols that cross the BBB [19], and their chemical properties, molecular conformations, and binding properties to Aβ42 or its aggregates are very similar to those of BOs [16,20]. To develop new strategies for AD treatment, we investigated in this study the interaction of Aβ42 and its aggregates with BDNF, examined the effects of these Aβ42 species on the cytological potency of extracellular BDNF, and explored the roles of BOs and CAs in these processes.

## 2. Results

### 2.1. Examination of the Binding Specificity of BDNF to Four Aβ42 Species

BDNF, a basic protein with an isoelectric point of 9–10, and Aβ42, a weakly acidic small-molecule protein (pI ≈ 5.3), exhibit inherent chemical interaction potential under physiological conditions. Different Aβ42 species (Aβ42M, Aβ42O, Aβ42P, and Aβ42F) exhibit distinct neurotoxic/neuroprotective effects on neuronal cell viability, and different BOs (e.g., IG3 and BCP) or CAs (e.g., CA and EGCG) have distinct binding specificities and affinities for various Aβ42 species [15,16,17]. Therefore, the binding rate of BDNF to each Aβ42 species was determined using indirect ELISA in the presence or absence of these modulators (IG3, BCP, CA, and EGCG), and the results were expressed as the binding specificity of BDNF for each Aβ42 species. HSA served as the negative control for the Aβ42 species.

**Figure 1:**
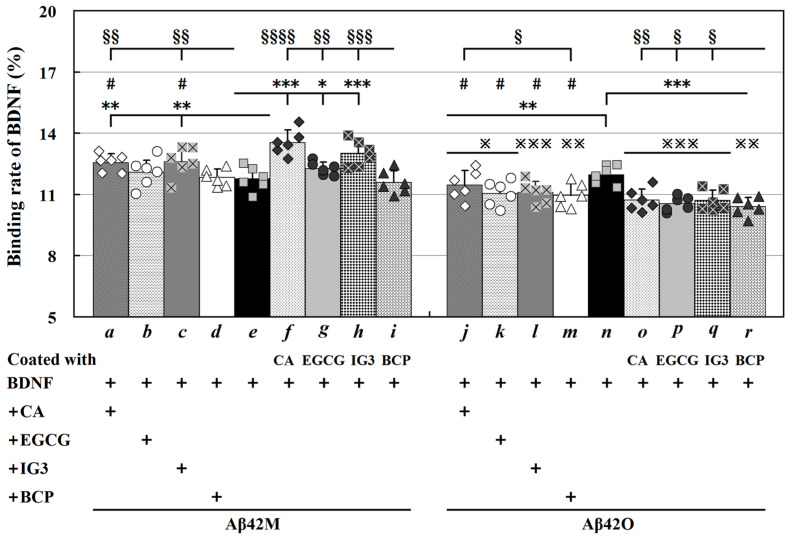
Binding rate of BDNF for Aβ42M and Aβ42O by indirect ELISA. The letters *a* through *r* indicate the corresponding groups. Binding rates of BDNF (final concentration: 20 pg/mL) to Aβ42M and Aβ42O (final concentration: 2.0 μM) with and without CA/EGCG/IG3/BCP (final concentration: 50 μM for CA/EGCG, 2.0 μM for IG3/BCP). The average amount of BDNF in the supernatants of the blank control groups was regarded as 100%. Each assay was performed in triplicate and repeated across six different batches of Aβ42. Values are presented as the mean ± SEM. Symbols *, #, §, and ※ indicate significant differences between groups with and without CA/EGCG/IG3/BCP, between groups with and without CA/EGCG/IG3/BCP in the coated Aβ42M/Aβ42O, between corresponding groups with CA/EGCG/IG3 and with BCP, and between corresponding Aβ42M and Aβ42O groups, respectively. */#/§/※ *p* < 0.05, **/§§/※※ *p* < 0.01, ***/§§§/※※※ *p* < 0.001, §§§§ *p* < 0.0001.

Under the conditions as shown in Figure 1, after 24 h of co-incubation, the binding rate of BDNF to HSA was less than 3% but it was greater than 10% for both Aβ42M and Aβ42O, indicating binding specificity between BDNF and Aβ42M/Aβ42O. However, BDNF showed minimal binding capabilities to larger Aβ42 aggregates, as indicated by a binding rate of less than 5% for both Aβ42P and Aβ42F. Furthermore, Figure 1 illustrates that the binding rate of BDNF to Aβ42O (*n* in Figure 1) was slightly higher than to Aβ42M (*e* in Figure 1), although this difference was not statistically significant within 24 h. The results for the Aβ42M group likely reflect the combined effects of Aβ42M and the newly formed Aβ42O generated by Aβ42M aggregation during the 24 h incubation period. This implies that BDNF had a higher binding specificity for Aβ42O than Aβ42M.

The results in Figure 1 show that the binding specificity of BDNF for Aβ42M and Aβ42O was affected differently by the four polyhydroxy compounds (IG3, BCP, CA, and EGCG). Overall, IG3, CA, and EGCG enhanced the binding rate of BDNF to Aβ42M (*a*, *c*, and *f*–*h*, Figure 1) (* *p* < 0.05, ** *p* < 0.01, or *** *p* < 0.001); however, all of these compounds, especially BCP and EGCG, decreased the binding rate of BDNF to Aβ42O (*j*–*m* and *o*–*r*, Figure 1) (** *p* < 0.01, or *** *p* < 0.001). These differing effects appear to result from the distinct binding properties of the compounds to Aβ42M because IG3 exhibits relatively high binding affinity for Aβ42M, whereas BCP shows relatively high binding affinity for Aβ42O [15].

Furthermore, when Aβ42M/Aβ42O was pre-incubated (co-coated) with these compounds for 12 h at 4 °C (*f*–*i* and *o*–*r*, Figure 1), the effect of these compounds on the binding rate (increase/decrease) of BDNF to Aβ42M/Aβ42O became more noticeable. However, the binding rate of BDNF to Aβ42M/Aβ42O remained unchanged regardless of the pre-incubation of the four compounds with BDNF. This implies that the compounds targeted Aβ42M/Aβ42O rather than BDNF, thereby altering the binding specificity of BDNF for Aβ42M/Aβ42O. Consequently, compared to the matching in-site systems co-incorporated with Aβ42M/Aβ42O and BDNF (*a*–*d* and *j*–*m*, Figure 1), these compounds had a more profound effect on Aβ42M/Aβ42O in their pre-incubated (co-coated) systems with Aβ42M/Aβ42O (*f*–*i* and *o*–*r*, Figure 1) (# *p* < 0.05). This suggests that the simultaneous binding of IG3/BCP/CA/EGCG and BDNF to an Aβ42 molecule or an Aβ42O unit was nearly impossible.

The results in Figure 1 indicate that IG3, CA, and EGCG increased the interaction between BDNF and Aβ42M most likely by preserving the native (or active) conformation of Aβ42 molecules, while they decreased the interaction between BDNF and an Aβ42O unit most likely by disrupting the integrated conformation of the Aβ42O unit according to previous findings [15]. Consequently, as Figure 1 illustrates, the binding rate of BDNF to Aβ42M was higher than that to Aβ42O when IG3, BCP, CA, or EGCG were present (※ *p* < 0.05, ※※ *p* < 0.01, or ※※※ *p* < 0.001). As BCP has a relatively low binding efficiency compared to Aβ42M [15], it had a minimal effect on BDNF binding to Aβ42M over 24 h. Indeed, BCP significantly increased the binding rate of BDNF to Aβ42M when the experiment duration was prolonged to 48 h. Thus, the results in Figure 1 suggest that additional extracellular substances, such as BOs or CAs, that interact with Aβ42M/Aβ42O may influence the binding specificity between extracellular BDNF and Aβ42M/Aβ42O.

### 2.2. Binding Affinity of BDNF for Aβ42M and Aβ42O

The specific binding between BDNF and Aβ42M/Aβ42O depends not only on specific, particularly polar interactions, but also on their conformation and the spatial arrangement or orientation of the interacting groups. These factors influence the strength and extent of the interaction force between them or their binding affinity. To assess the binding affinity of BDNF for Aβ42M and Aβ42O, the equilibrium dissociation constants (*K*_D_) of BDNF with Aβ42M and Aβ42O in the presence and absence of CA, EGCG, IG3, and BCP, respectively, were determined using indirect competitive ELISA and were then used to inversely express the binding affinity of BDNF for Aβ42M and Aβ42O.

The amount of BDNF in the well that was not bound to the immobilized Aβ42M/Aβ42O was measured using the anti-BDNF antibody after the establishment of equilibrium relationships of BDNF between immobilized and free Aβ42M/Aβ42O were established. The results are shown in the supplementary materials (Figure S1).

The values on these curves corresponding to the 50% maximum absorbance were used to calculate the *K*_D_ values for each group. As shown in Table 1, the *K*_D_ value of BDNF for Aβ42M (6.67 × 10^−7^ M) was marginally higher than that for Aβ42O (2.88 × 10^−7^ M), indicating that the binding affinity between BDNF and Aβ42O was marginally higher than that between BDNF and Aβ42M, and vice versa. Combined with the results in Figure 1, which show that the binding specificity of BDNF for Aβ42O was higher than that for Aβ42M (*n* and *e*, Figure 1), it could be concluded that BDNF and Aβ42O interacted or bound more strongly and stably than BDNF and Aβ42M. In contrast, BDNF dissociates from Aβ42M more quickly and easily than from Aβ42O.

Furthermore, the *K*_D_ values of BDNF for Aβ42M and Aβ42O changed in opposite directions when CA, EGCG, IG3, or BCP was present; specifically, the *K*_D_ values decreased for Aβ42M and increased for Aβ42O (Table 1). The lowest *K*_D_ values were observed in the Aβ42M with IG3 group (3.89 × 10^−7^ M), whereas the highest was in the Aβ42O with BCP group (2.34 × 10^−5^ M), respectively. According to these findings, the polyhydroxy compounds increased the binding affinity of BDNF for Aβ42M (particularly with IG3) while reducing the binding affinity of BDNF for Aβ42O (particularly with BCP). This implies that these compounds enhanced the interaction between BDNF and Aβ42M while blocking or reducing the interaction between BDNF and Aβ42O. These results can be explained as follows: for Aβ42M, these compounds preserved or maintained the conformation of Aβ42M, which increased the binding affinity between Aβ42M and BDNF in addition to improving the binding efficiency between them; for Aβ42O, they blocked or disrupted the integrated conformation of an Aβ42O unit, which decreased the binding affinity between Aβ42O and BDNF as well as the binding efficiency between them. The BDNF-Aβ42M complex should have a significantly smaller interface area than the BDNF-Aβ42O complex because Aβ42 is a small-molecule protein. This could make the BDNF-Aβ42M complex easier to dissociate than the BDNF-Aβ42O complex.

The binding affinity between BDNF and Aβ42M/Aβ42O was significantly higher than that of BOs and Aβ42M/Aβ42O as the *K*_D_ values of BOs for Aβ42M/Aβ42O were 10- to 1000-fold higher than those of BDNF for Aβ42M/Aβ42O) [15], and was higher than that between Aβ42O and human anti-Aβ42O single-chain antibodies, whose *K*_D_ values of the antibodies were roughly 10-fold higher than those of BDNF for Aβ42O [21,22,23,24].

### 2.3. Differential Effects of Aβ42M and Aβ42O on BDNF-Supported Neural Cell Survival

It is well established that the viability or survival of neuronal cells in vitro and in vivo is directly correlated with the neurotoxicity of Aβ42O and the neurotrophicity of Aβ42M. We have shown that BOs (such as IG3 and BCP) have strong binding capabilities for both Aβ42M and Aβ42O and either protect or enhance neuronal cell viability in the presence of Aβ42M and Aβ42O [15]. However, the results in Table 1 indicate that the binding affinity of BDNF for Aβ42M and Aβ42O is higher than that of BOs and that CA/EGCG/IG3/BCP also modulate the binding affinity of BDNF as well.

As physiological BDNF levels in the CNS and in SH-SY5Y and HT22 cultures are extremely low (picogram levels) [25,26,27], even slight fluctuations in BDNF levels may critically influence neuronal cell survival. To determine whether Aβ42M and Aβ42O influence the supportive efficacies of BDNF on neuronal cell survival and whether CA/EGCG/IG3/BCP preserves or potentiates the cytological functions of BDNF via Aβ42M and Aβ42O, we systematically examined the combinatorial effects of these substances or anti-BDNF antibody (Ab) with Aβ42M and Aβ42O on the viability of SH-SY5Y and HT22 cells under distinct intervention sequences.

**Figure 2:**
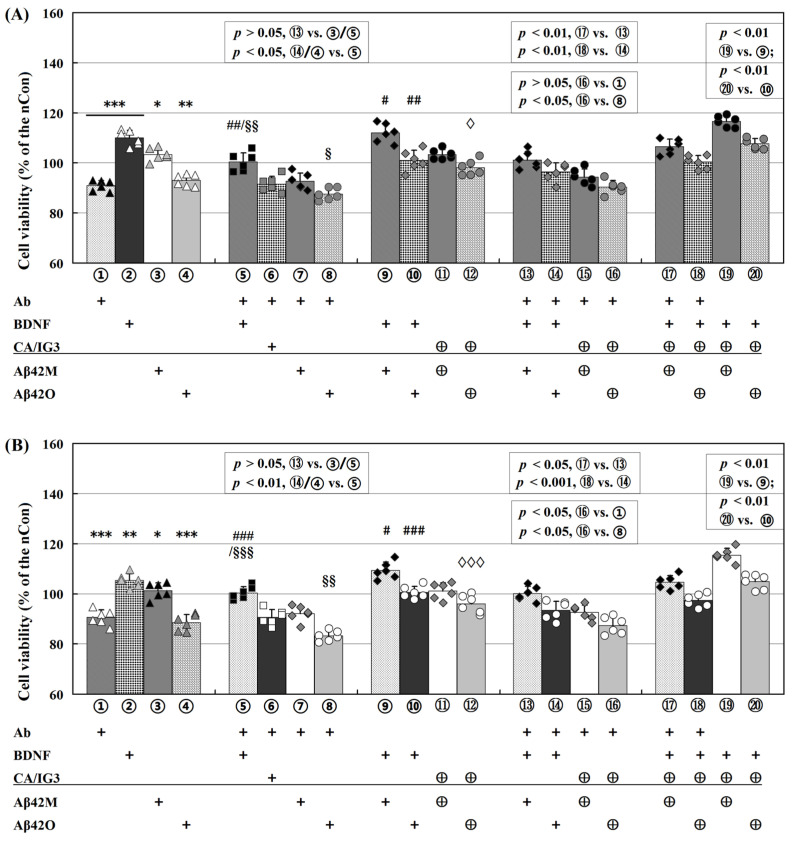
Differential effects of Aβ42M and Aβ42O on BDNF-supported neural cell survival. Viability of SH-SY5Y (**A**) and HT22 (**B**) cells in the presence of anti-BDNF antibody (Ab), BDNF, and Aβ42M/Aβ42O with and without CA and IG3 for 2 h at 37 °C quantified by MTT assay. The viability of the blank control group was defined as 100%. Symbol ⊕ indicates addition after being pre-incubated for 0.5 h at 4 °C. Symbols *, #, §, and ◊ indicate significant differences between the experimental and control groups, between BDNF alone and other groups, between Ab alone and other groups, and between Aβ42O alone and other groups, respectively. */#/§/◊ *p* < 0.05, **/##/§§ *p* < 0.01, and ***/###/§§§/◊◊◊ *p* < 0.001.

When compared to their respective controls (100%), CA/EGCG/IG3/BCP alone did not affect the viability of SH-SY5Y or HT22 cells. However, Ab and BDNF alone significantly decreased (down to ~91%) and increased (up to ~110%) the viability, respectively (① and ②, Figure 2A,B). Meanwhile, the viability under the co-treatment with Ab/BDNF and CA/IG3 (⑥, Figure 2A), other was identical to that with Ab or BDNF alone. These results imply that the viability of these neuronal cells was positively correlated with the level of free BDNF, either endogenous or exogenous BDNF, and that an insufficient amount of BDNF in the culture medium directly reduced the cell viability. Additionally, the cell viability in the group co-treated with Ab and BDNF (⑤, Figure 2A) was identical to that of the control group, indicating that Ab and exogenous BDNF had equivalent effectiveness under these experimental settings.

As shown in Figure 2A (SH-SY5Y cells), the neurotrophic effect of Aβ42M on the cell viability appeared to depend on the BDNF level (③, ⑦, ⑨, and ⑬, Figure 2A). The corresponding groups with and without Aβ42M did not differ in cell viability while Ab was present (*p* > 0.05, ① vs. ⑦/⑤ vs. ⑬, Figure 2A). However, the effect of Aβ42M became more apparent as the BDNF levels increased (*p* < 0.05, ③ vs. control/② vs. ⑨, Figure 2A), suggesting that Aβ42M enhanced the BDNF efficacy. These results indicate a synergistic effect of Aβ42M on BDNF, i.e., the binding of Aβ42M to BDNF might enhance the cytological efficacy of BDNF in targeting and binding to cells. This suggests that the neurotrophic effects of Aβ42M might be primarily related to its synergistic effects on BDNF. This could be the cause of the dependence of Aβ42M’s neurotrophic effects on the BDNF levels. Additionally, the synergistic effect of Aβ42M on BDNF was more significant after pre-incubating with CA or IG3 (*p* < 0.01, ⑨ vs. ⑲/⑰ vs. ⑬, Figure 2A). However, there was no difference in the cell viability between the Aβ42M alone group and Aβ42M with CA/IG3 groups when the BDNF level was low (③ and ⑪, ⑦ and ⑮, Figure 2A). These findings were consistent with the results showing that BOs and CAs facilitated the binding between Aβ42M and BDNF (Figure 1 and Table 1).

In contrast, Aβ42O continued to impair the cell viability in the presence of Ab, suggesting that Aβ42O-induced neurotoxicity was somewhat dependent on the BDNF levels (*p* < 0.05, ① vs. ⑧/⑤ vs. ⑭, Figure 2A). However, as the BDNF level increased, the damage caused by Aβ42O to the cell viability was progressively reduced (④ and ⑩, Figure 2A), which was inevitable because Aβ42O and BDNF bound together (Figure 1 and Table 1). These results imply that a substantial portion of the neurotoxicity of Aβ42O was caused by a reduction in BDNF effectiveness rather than merely a mix of the individual impacts of Aβ42O and BDNF on the cell viability. Furthermore, regardless of whether these impairments were related to BDNF, the binding of CA/IG3 to Aβ42O (during pre-incubation) significantly decreased the impairment of cell viability caused by Aβ42O (*p* < 0.05, ⑧ vs. ⑯/④ vs. ⑫, Figure 2A). Regarding the BDNF-related Aβ42O neurotoxicity, the protective effect of CA/IG3 on BDNF against Aβ42O appeared to be positively correlated with BDNF levels [(*p* < 0.05, ⑧ vs. ⑯; *p* < 0.01, ⑱ vs. ⑭) or (*p* < 0.05, ④ vs. ⑫; *p* < 0.01, ⑩ vs. ⑳), Figure 2A].

The results shown in Figure 2B and the results mentioned above were also confirmed in HT22 cells. Overall, the results of HT22 cells were almost identical to those of SH-SY5Y cells, with some minor differences. In HT22 cells, the synergistic effect of Aβ42M on BDNF was comparable to or marginally weaker than that in SH-SY5Y cells (③, ⑨, and ⑬, Figure 2B), whereas the toxic effect of Aβ42O, regardless of its relation to BDNF, was marginally stronger than that in SH-SY5Y cells (④, ⑩, and ⑭), Figure 2B). These patterns were observed in how CA/IG3 influenced Aβ42M’s synergistic effect on BDNF (⑪, ⑰, and ⑲, Figure 2B) and Aβ42O’s toxic effect (⑫, ⑱, and ⑳, Figure 2B). This suggests that the effects of Aβ42M or Aβ42O on the cytological efficacy of BDNF, as reflected in the cell viability or survival, were identical across different neuronal cell systems.

Furthermore, EGCG and BCP were systematically substituted for CA and IG3 within the established protocol. Quantitative analyses indicated that the neuroprotective effects were comparable to those in Figure 2, with BCP demonstrating a marginally enhanced cytoprotective effect against Aβ42O relative to other test compounds. Furthermore, dose–response characterization demonstrated concentration-dependent therapeutic effects across all investigated agents: CA and EGCG exhibited optimal activity within the 0–100 μM ranges, while IG3 and BCP showed maximal efficacy at 0–2.0 μM concentrations.

Taken together, the results in Figure 2 suggest that the beneficial effect of Aβ42M and the detrimental effect of Aβ42O on neuronal cell viability or survival were substantially mediated by their binding to BDNF. A significant part, if not all, of the mechanism underlying the neurotrophicity of Aβ42M and the neurotoxicity of Aβ42O appeared to involve increased Aβ42M, but Aβ42O decreased the supportive efficacy of BDNF on neuronal cell survival through their specific binding to BDNF.

### 2.4. Examination of the Sites and Properties of the Interaction Between Aβ42M/Aβ42O and BDNF by Molecular Docking

Our previous studies have shown that both BOs bind to the Aβ42 chain(s) axially or in a forked form [15]. However, BDNF also binds to Aβ42M and Aβ42O with greater affinity than BOs and CAs (Figure 1 and Table 1). To elucidate the mechanistic effects of distinct molecular interactions on the cytological function of BDNF and establish their correlations, we conducted systematic molecular docking analyses between BDNF and three critical targets: Aβ42M, Aβ42 trimer, and its receptor TrkB. These computational findings were subsequently compared with the integrated conformation of the NT4-TrkB reference complex.

**Figure 3:**
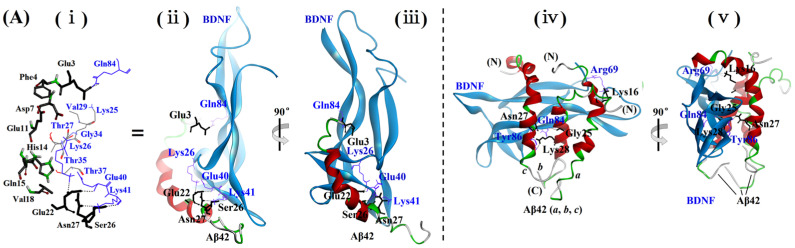

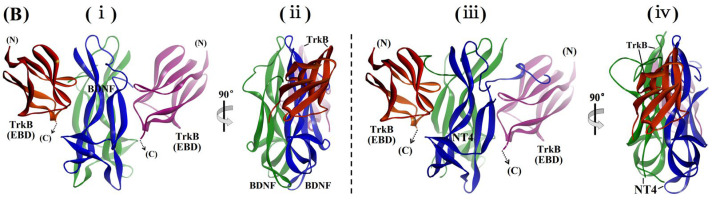
Molecular docking models of BDNF to Aβ42M, Aβ42 trimer, and the extracellular binding domain of TrkB. (**A**) Docking models of BDNF to Aβ42M chain and Aβ42 trimer (peptide chains were marked with symbols *a*, *b*, and *c*, respectively), showing the intermolecular hydrogen bonds (dashed lines) between BDNF’s (lines) and the Aβ42 trimer’s (stick) amino acid residues. (**i**) main amino acid residues involved in the interaction between BDNF and Aβ42; (**ii**,**iii**) docking model of BDNF with Aβ42; (**iv**,**v**) docking model of BDNF with Aβ42 trimer. (**B**) Docking models of BDNF to the extracellular binding domain (EBD) of TrkB (**i**,**ii**) and the crystal structure of NT4-TrkB complex downloaded from PDB (ID: 1HCF) (**iii**,**iv**). N or C: N or C terminus of Aβ42 chain.

Docking models of BDNF and Aβ42M (Figure 3A(ii,iii)) showed that only the bottom portion of the BDNF molecule bound to the N-terminal (1–16 residues) and middle (17–28 residues) regions of the Aβ42 molecule. This suggested that the interactions between polar or hydrophilic groups on the main and side chains in their interfacial regions—with or without the formation of H-bonds—were the main forces driving and stabilizing their binding (Figure 3A(i)). Furthermore, the docking model of BDNF and Aβ42M showed that the binding between Aβ42M and BDNF did not appear to spatially affect the binding of BDNF to the extracellular binding domain of its receptor TrkB, nor did it produce a spatial barrier to the dimerization of BDNF for the stable binding of BDNF to TrkB, as demonstrated in Figure 3B. This implies that the combination of Aβ42M and BDNF might stabilize the conformation of BDNF and enhance its potency without diminishing its cytological effects. Combined with the beneficial effects of Aβ42M on cell viability (③ and ⑨, Figure 2A,B), the BDNF-Aβ42M docking model suggests that Aβ42M binding to BDNF facilitated or enhanced the efficiency of BDNF to target and bind to its receptors in the cellular system. Thus, the apparent depletion of BDNF by Aβ42O may be primarily due to their unique binding pattern in addition to their high binding affinity. This might be the mechanism underlying the synergistic effect of Aβ42M on BDNF.

In contrast, the binding between Aβ42O and BDNF was stronger and more stable than that between Aβ42M and BDNF, as demonstrated by the docking model of BDNF with Aβ42O (Figure 3A(iv,v)), which also revealed that the interaction sites and interface area between Aβ42O and BDNF were substantially greater than those between Aβ42M and BDNF. This finding explains why the binding performance of BDNF to Aβ42O was higher than that for Aβ42M (Figure 1 and Table 1). Additionally, nearly the entire flank of BDNF—specifically, the interface where it dimerizes (Figure 3B(i,ii))—was involved in the binding between Aβ42 trimer and BDNF (Figure 3A(iv,v)). This suggests that BDNF was rendered inactive, inhibiting its biological activity by Aβ42O binding, which lowered the level of effective BDNF. This is evidenced by the reduced cell viability observed in the Aβ42O groups containing either endogenous or exogenous BDNF, as shown in Figure 2A,B (③ and ⑨). Compared with the electrostatic energy (Eelec) (−428.5 kcal/mol) of the Aβ42 trimer-BDNF complex (Table S1), the slightly higher Eelec (−413.0 kcal/mol) of the Aβ42M-BDNF complex suggests that the Aβ42M-BDNF complex might have slightly lower stability than the Aβ42 trimer-BDNF complex. This was consistent with the difference in their binding affinities, as shown in Table 1.

Furthermore, the regions on the Aβ42M chain and Aβ42O unit where BDNF was bound were similar to or overlapped with those where IG3/BCP was bound to an Aβ42M chain or Aβ42O unit [15], particularly on the Aβ42M chain, as indicated by the docking models shown in Figure 3A. This suggests that competition between BDNF and BOs/CAs for binding to Aβ42M or Aβ42O is inevitable. Consequently, in the presence of both BDNF and BOs/CAs, their binding efficiency to Aβ42M or Aβ42O depended on their concentration and binding affinity to Aβ42M or Aβ42O, similar to the interaction between an enzyme’s substrate and its competitive inhibitor. This was confirmed by the corresponding results for the Aβ42M and Aβ42O groups shown in Figure 2A,B (⑪–⑫ and ⑲–⑳). Combined with the results in Figure 2A,B, it could be concluded from the docking models in Figure 3A that BOs/CAs bound to Aβ42M were easily displaced by BDNF, whereas those bound to Aβ42O were not. As a result, only a fraction of BOs/CAs in the Aβ42O groups shown in Figure 2A,B might be displaced by BDNF.

BDNF and NT4 share the same receptors on neuronal cells and bind to them in a similar pattern [28]. BDNF and NT4 are structurally related proteins, sharing more than 50% identity in their amino acid sequences; therefore, their three-dimensional (3D) structures have very similar features. The docking model of BDNF with the extracellular binding domain of TrkB shown in Figure 3B suggests that the docked conformations of the BDNF-TrkB complex (Figure 3B(i,ii)) closely matched those of the crystal structure of the NT4-TrkB complex (Figure 3B(iii,iv)). Therefore, the 3D structural model of the BDNF-TrkB complex is credible.

### 2.5. Effects of Aβ42M and Aβ42O on the Receptor-Targeting Performance of BDNF in the Presence and Absence of IG3/BCP/CA/EGCG by IF Staining

The supporting effects of BDNF on neuronal cell survival are mediated by its two receptors: the high-affinity TrkB and low-affinity p75. The activation of this coordinated BDNF/TrkB/p75 signaling axis plays a critical role in maintaining neuronal viability and regulating synaptic plasticity. Despite the characteristically low basal levels of BDNF in the CNS and the relatively sparse membrane expression of TrkB and p75 receptors under physiological conditions [25], the binding affinity between BDNF and TrkB/p75 is significantly higher than that between BDNF and Aβ42M/Aβ42O according to their extremely low dissociation constants of 10^−11^ mol/L and 10^−9^ mol/L for TrkB and p75, respectively [29]. Our earlier studies have demonstrated that both Aβ42M and Aβ42O can target cells [8], but neither colocalized with TrkB or p75 in our preliminary IF experiments. Given the effects of Aβ42M and Aβ42O on BDNF-related cell viability (Figure 2) and the distinct binding patterns of BDNF in the Aβ42M-BDNF and Aβ42O-BDNF complexes (Figure 3A), it is crucial to determine whether Aβ42M and Aβ42O (with or without IG3/BCP/CA/EGCG) influence the targeting and binding of BDNF to TrkB/p75 at the SH-SY5Y plasma membranes. This is essential for clarifying the mechanisms by which different Aβ42 species affect neuronal cells.

**Figure 4:**
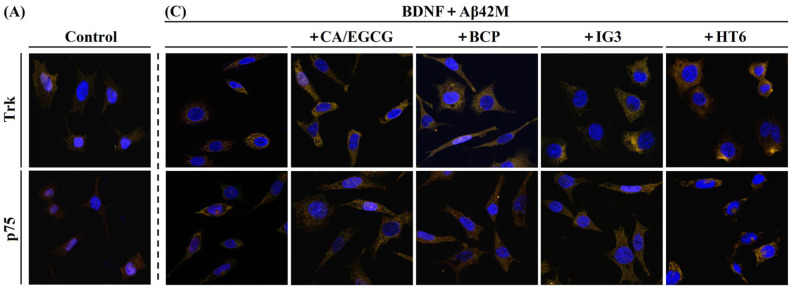

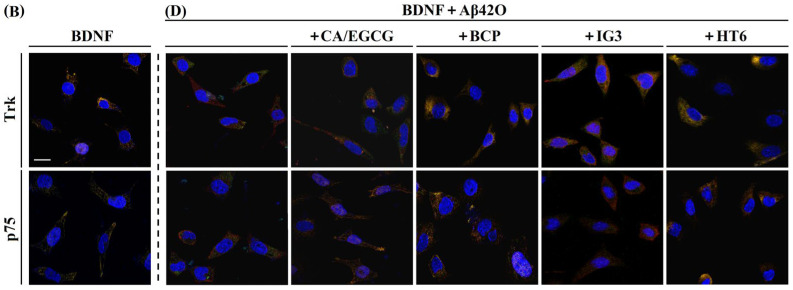
Representative confocal images of double-labeling immunofluorescence of BDNF (green) and TrkB/p75 (red) in SH-SY5Y cells. The merged images include BDNF (green), TrkB/p75 (red), nucleus (blue), and the BDNF-TrkB/p75 complex (yellow). (**A**) Double-IF images of control group. (**B**) Double-IF images of BDNF group. (**C**,**D**) Double-IF images of Aβ42M and Aβ42O groups with or without IG3/BCP/CA/EGCG/HT6, respectively. The first and second rows in (**A**–**D**) represent TrkB and p75 groups, respectively. Scale bar = 20 μm.

The colocalization of the extracellular BDNF with the membrane TrkB/p75, which can be identified at the cellular level via IF staining, indicates their binding on the plasma membrane. Figure 4 shows representative images of SH-SY5Y cells in which BDNF and membrane TrkB/p75 were fluorescently labeled with specific anti-BDNF (green) and anti-TrkB/p75 (red) antibodies, respectively. Their spatial overlap at the same pixel was considered evidence of colocalization (yellow). Overall, in all four groups (Figure 4A–D), BDNF colocalized more with TrkB than with p75, indicating a positive correlation with BDNF affinity for both receptors. Compared to the levels of BDNF colocalization with TrkB and p75 in the BDNF group without Aβ42M or Aβ42O (Figure 4B), the levels of BDNF colocalization with TrkB or p75 appeared to be elevated in the Aβ42M group (Figure 4C, column 1), whereas they were reduced in the Aβ42O group (Figure 4D, column 1). These phenomena suggest that Aβ42M and Aβ42O had specific but opposite effects on the binding of BDNF to the receptors; Aβ42M promoted binding, while Aβ42O inhibited it. The parameters MOC and the colocalization coefficient M2 (for BDNF) quantitatively confirmed these results (group 1, 2, 9, 14, 19, and 24, Table 2). This supports our conclusion from the Aβ42M and Aβ42O docking models with BDNF (Figure 3A) that Aβ42O inhibits BDNF’s ability to bind to its receptors, whereas Aβ42M does not. More specifically, by binding to BDNF in a different way (Figure 3A), Aβ42M might function to synergistically provide BDNF for binding to its receptors by binding to BDNF differently than Aβ42O, which instead consumed BDNF. This was consistent with the result that Aβ42O had a slightly higher affinity for BDNF than Aβ42M (Table 1). Clearly, the impacts of Aβ42M and Aβ42O on the colocalization of BDNF with its receptors were consistent with their effects on BDNF-related neuronal cell viability (③, ④, ⑨, ⑩, Figure 2A,B). Additionally, the colocalization coefficient M1 (for TrkB/p75) of the Aβ42M group was marginally lower than that of the BDNF group (Table 2). This suggests that the presence of Aβ42M increased the binding effectiveness of BDNF to its receptors, which in turn increased cell survival (*p* < 0.05, ③ vs. control/⑨ vs. ②, Figure 2A,B).

Furthermore, BDNF colocalization with both receptors was significantly increased in the Aβ42M or Aβ42O groups when IG3/BCP/CA/EGCG/HT6 were present (Figure 4C,D), especially in the HT6 subgroups. In contrast, BDNF colocalization with TrkB or p75 remained unchanged when IG3/BCP/CA/EGCG/HT6 were present without Aβ42M/Aβ42O. These findings demonstrated that IG3/BCP/CA/EGCG competitively influence Aβ42M or Aβ42O binding to BDNF, thereby enhancing BDNF binding to its receptors. HT6, an scFv anti-Aβ42O antibody, reduced Aβ42O binding to BDNF by inducing the integrated conformation of the (freshly formed) Aβ42O units to be disrupted [24]. As listed in Table 2, the MOC, M1, and M2 parameters values in the Aβ42M/Aβ42O groups with IG3/BCP/CA/EGCG/HT6 (groups 5–8, 10–13, 15–18, and 20–23) were higher than those in the Aβ42M/Aβ42O group without those compounds (group 9, 14, 19, and 24), and in the presence of IG3/BCP/CA/EGCG/HT6 alone, these parameter values were almost identical to those of the control group. This finding indicates that these substances increased either the proportion of BDNF bound to its receptors or the amount of BDNF-TrkB/p75 complexes on the plasma membrane of neuronal cells. This may be because these substances competitively bound to Aβ42M and Aβ42O, increasing the Aβ42M presentation of BDNF to its receptors and/or decreasing the Aβ42O consumption of BDNF. This effect could also enhance BDNF’s sensitivity to its receptor and increase its binding rate. Therefore, IG3/BCP/CA/EGCG had consistent impacts on the role of Aβ42M/Aβ42O in BDNF binding to its receptors (Figure 4, Table 2) and on neuronal cell viability or survival (Figure 2).

### 2.6. Effects of Aβ42M and Aβ42O on BDNF Binding to TrkB/p75 in the Presence and Absence of IG3/BCP/CA/EGCG by Co-IP

**Figure 5:**
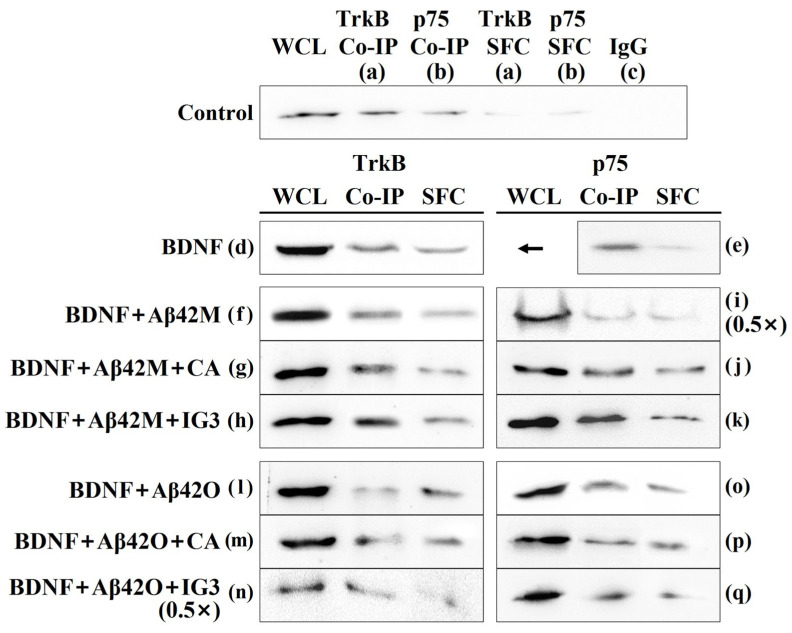
Detection of BDNF bound to TrkB and p75 by Western blot analysis after Co-IP. Co-IP was conducted using anti-TrkB and anti-p75 antibodies, with rabbit IgG serving as a negative control. BDNF bound to TrkB and p75 was detected using an anti-BDNF antibody. The symbols indicating groups in Figure 5 correspond to those listed in Table 3. The letters a through q indicate the corresponding groups. WCL: whole-cell lysate samples; SFC: supernatants from Co-IP; Co-IP: Co-IP beads. SFC and Co-IP samples were prepared from equal amounts of WCL within the same group, while the WCL samples were obtained from lysates of initial cells equivalent to those of the control groups. The symbol 0.5× indicates that half of the sample size was used. An arrow in subgroup e indicates that the WCL sample in the p75 group is the same as that in the TrkB group.

To further confirm the impact of Aβ42M and Aβ42O on BDNF binding to TrkB/p75 in the presence and absence of IG3/BCP/CA/EGCG, BDNF bound to TrkB/p75 was then identified by a Co-IP assay using an anti-TrkB/p75 antibody, followed by Western blotting with an anti-BDNF antibody. Figure 5 illustrated that only the WCL, SFC, and Co-IP groups treated with the anti-BDNF antibody exhibited a BDNF signal (a and b in Figure 5), whereas the control IgG group did not (c in Figure 5) (control IgG data for all other experimental groups were not presented). Additionally, BDNF was detected in both Co-IP and SFC samples, confirming that it binds to TrkB and p75 receptors simultaneously. Immunoblot densities in Figure 5 were analyzed and BDNF levels were quantified, as presented in Table 3.

The comparison of BDNF signals in the control (a and b in Figure 5 and Table 3) and BDNF (d and e in Figure 5 and Table 3) groups indicated that the degree of BDNF co-precipitated with TrkB and p75 increased as the BDNF level in the culture medium increased, revealing a positive correlation between the amount of BDNF bound to the receptors and the amount of free BDNF in the extracellular matrix. Overall, the Aβ42M group (f and i in Figure 5 and Table 3) showed greater BDNF co-precipitation with TrkB or p75 than the control BDNF group, while the Aβ42O group showed less (l and o in Figure 5 and Table 3). These results confirmed that Aβ42M promoted BDNF binding to both receptors, whereas Aβ42O inhibited it. Apparently, the Co-IP results were consistent with those of IF (Figure 4 and Table 2).

Based on the binding specificity of Aβ42M and Aβ42O for BDNF (Figure 1) and preliminary IF results showing that they are neither colocalized with TrkB or p75, it is evident that Aβ42M and Aβ42O exerted opposite effects on BDNF-receptor binding due to their specific binding to BDNF rather than to the receptors. Given the differences in the binding strength between Aβ42M and Aβ42O to BDNF (Figure 3A) and the lower total BDNF levels in the WCL samples of the Aβ42O group (l and o in Table 3) compared to the BDNF group (d and e in Table 3), it is suggested that Aβ42M increases the binding rate of BDNF to its receptors by binding more reversibly to extracellular BDNF, whereas Aβ42O may consume BDNF by binding less reversibly to extracellular BDNF. Here, the action of Aβ42M enhanced the sensitivity or targeting of BDNF to its receptors, effectively synergizing BDNF binding and enhancing the attachment of BDNF to its receptors. The Aβ42O consumption of BDNF may be explained by the stronger chemical and physical (or stacking) interactions between BDNF molecules and Aβ42 chains in the Aβ42O-BDNF complex (Figure 3A), which likely prevented BDNF from being (fully) released in the Co-IP experiment and prevented recognition by the anti-BDNF antibody. This indicated that the amount or efficiency of BDNF binding to its receptors depends on the level of free or effective BDNF, rather than total BDNF, in the extracellular matrix. Thus, BDNF molecules in Aβ42M-BDNF complexes may be regarded as effective BDNF molecules, that is, BDNF molecules that attach to their receptors favorably, whereas those BDNF molecules in the Aβ42O-BDNF complexes may be considered as ineffective or consumed.

Furthermore, both the Aβ42M (g, h and j, k in Figure 5 and Table 3) and Aβ42O (m–n and p, q in Figure 5 and Table 3) groups showed increased BDNF co-precipitation with TrkB/p75 in the presence of CA and IG3, whereas the results in our preliminary experiments showed that CA/IG3 alone did not have any effect on either the TrkB/p75 levels or co-precipitation of BDNF with TrkB/p75. In addition, EGCG and BCP showed effects corresponding to those of CA and IG3, respectively, on the co-precipitation of BDNF with TrkB/p75.

This should be attributed to the dynamics of Aβ42M/Aβ42O binding to CA/EGCG/IG3/BCP and BDNF or the competition between CA/EGCG/IG3/BCP and BDNF for their binding to Aβ42M or Aβ42O. Consequently, CA/EGCG/IG3/BCP increased the effectiveness of Aβ42M in presenting BDNF to its receptors while reducing the Aβ42O consumption of BDNF. It was clear that all binding was related to the concentration of the chemicals involved in these processes.

## 3. Materials and Methods

### 3.1. Aβ42 Species and Other Proteins

Human Aβ42 protein was purchased from Dalian Meilun Biological Co., Ltd. (MB10425, Dalian, China), and its purity was higher than 96%. Four Aβ42 species (Aβ42M, Aβ42O, Aβ42P, and Aβ42F) were prepared as described previously [3] and confirmed by electron microscopy and fluorescence spectroscopy based on the binding of thioflavin T (ThT) as described previously [21,30]. For ThT assay, the fluorescence intensities of Aβ42O, Aβ42P, and Aβ42F (each 5.0 μM) were typically about 230 ± 20, 350 ± 26, and 500 ± 30, respectively, corresponding to their states observed in electron microscopy as previously reported [21]. HT6 was obtained as described previously [24]. Human-brain-derived neurotrophic factor (BDNF), anti-BDNF antibody, and anti-TrkB antibody were purchased from Shanghai Abcam Trading Co., Ltd. (ab222178/ab108319/ab134155, Shanghai, China). BF488-conjugated anti-BDNF antibody (green) and Goat anti-mouse/rabbit IgG H&L/HRP were purchased from Beijing Biosynthesis Biotechnology Co., Ltd. (bs-4989R-BF488/bs-0296G-HRP/bs-0295G-HRP, Beijing, China). Anti-p75 antibody and Rabbit IgG were purchased from Shanghai Beyotime Biotechnology Co., Ltd. (AF1033/A7016, Shanghai, China). AF594-conjugated anti-TrkB antibody (red) and AF647-conjugated anti-p75 antibody (red) were purchased from Shanghai Santa Cruz Biotechnology Co., Ltd. (sc-7268 AF594/sc-271708 AF647, Shanghai, China). Protein A&G agarose beads was purchased from Shanghai Roche Co., Ltd. (11719408001, Shanghai, China). Prestained protein marker was purchased from Wuhan Servicebio Technology Co., Ltd. (G2058, Wuhan, China). Human serum albumin (HSA) was purchased from Beijing Solarbio Science and Technology Co., Ltd. (A8230, Beijing, China).

### 3.2. Chemicals

CA, EGCG, and IG3 were purchased from Shanghai Yuanye Biochemical Technology Co., Ltd. (B21722/B20106/B27498, Shanghai, China). BCP was purchased from Shanghai SCR-Biotech Co., Ltd. (SCGC-101239, Shanghai, China). These oligosaccharides are in D-type configuration. Unless otherwise noted, all solutions were prepared using double distilled water (ddH_2_O). Their stock solutions were prepared to a concentration of 1.0 mM in ddH_2_O. DAPI solution was purchased from Beijing Biosynthesis Biotechnology Co., Ltd. (C02-04003, Beijing, China). Native lysis buffer was purchased from Beijing Solarbio Science and Technology Co., Ltd. (R0030, Beijing, China). BeyoECL Star Kit was purchased from Shanghai Beyotime Biotechnology Co., Ltd. (P0018AS, Shanghai, China). All other chemicals were local products of analytical grade.

### 3.3. Cell Culture

In this study, human neuroblastoma cell line SH-SY5Y and mouse primary hippocampal neuronal cell line HT22 (100158/358041, BeNa Culture Collection, Beijing, China) were used as model cell lines for neural cells. Unless otherwise stated, the cells were cultured in Dulbecco’s modified eagle medium (DMEM) (11965092, GIBCO, Shanghai, China) with 10% fetal bovine serum (FBS, 16140071, GIBCO, Shanghai, China) and 100 U/mL penicillin–100 µg/mL streptomycin (15140148, GIBCO, Shanghai, China) in a humidified atmosphere of 5% CO_2_ at 37 °C (standard conditions) for a specified period of time. All experiments were performed between the 3rd and 6th passages.

### 3.4. Determination of the Binding Specificity of BDNF to Different Aβ42 Species by Indirect Enzyme-Linked Immunosorbent Assay (ELISA)

#### 3.4.1. Construction of a Standard BDNF Curve

Seven samples of BDNF aqueous solutions (0 to 80 pg/mL) were prepared in ELISA coating buffer (C1050, Beijing Solarbio Technology Co., Ltd., Beijing, China) and coated onto 96-well plates (100 μL per well) at 4 °C for 12 h. After aspirating the liquid, the protein content in the aspirated coating solution was determined to ensure that BDNF was completely coated on the plate. All coated BDNF was then assayed by conventional indirect ELISA using anti-BDNF antibody and goat anti-mouse IgG H&L/HRP antibody at 37 °C for 1.5 h. Finally, the absorbance of each well was measured at 450 nm on a microplate reader. The measurement was performed in triplicate. A standard curve of BDNF concentration (pg/mL) was plotted using the averaged data, yielding an equation of *y* = 0.0002 *x* + 0.0013 with a correlation coefficient of 0.9687.

#### 3.4.2. Determination of the Binding Rate of BDNF to Different Aβ42 Species

Four Aβ42 species (Aβ42M, Aβ42O, Aβ42P, and Aβ42F) and HSA (each at a final concentration of 2.0 μM) or their respective mixtures with CA/EGCG/IG3/BCP (final concentration: 50 μM for CA/EGCG, 2.0 μM for IG3/BCP) were coated onto 96-well plates and incubated at 4 °C for 12 h. PBS (10 mM, pH 7.4) was used as a blank control. The coating solution was discarded after confirming the complete coating of each protein on the plate, and the coated wells were blocked with blocking solution [5% nonfat dry milk in TBS buffer (10 mM Tris-HCl, pH 7.5, 75 mM NaCl)] (150 μL per well) for 1 h at room temperature (21 °C). Then, 100 μL of BDNF solution (final concentration 20 pg/mL) with or without CA, EGCG, IG3, or BCP (final concentration: 50 μM for CA/EGCG, 2.0 μM for IG3/BCP) was added to each coated well, and the plate was incubated at 37 °C for 24 h. The supernatant was aspirated, and the amount of BDNF bound in each well and the amount of BDNF in the supernatant from blank control wells were detected using anti-BDNF antibody by indirect ELISA as described above. Measurements for every experiment were performed in triplicate and repeated across six independent Aβ42 batches.

The binding specificity of BDNF for different Aβ42 species was expressed as its binding rate, determined from the standard curves for BDNF concentrations. The binding rate of BDNF for different Aβ42 species or HSA was calculated by taking the difference between the mean data of the Aβ42/HSA-coated wells and blank control wells (uncoated with Aβ42), dividing by the amount of BDNF in 100 μL of BDNF solution above and multiplying the result by 100%.

### 3.5. Measurement of the Equilibrium Dissociation Constant (K_D_) of BDNF to Aβ42M/Aβ42O by Indirect Competitive ELISA

The equilibrium dissociation constant (*K*_D_) of BDNF to Aβ42M/Aβ42O represented the binding affinity between BDNF and Aβ42M/Aβ42O, which was measured by indirect competitive ELISA. Aβ42M/Aβ42O at Aβ42 concentration of 10^−12^ to 10^−5 M^ were mixed with BDNF (final concentration 20.0 pg/mL) or with BDNF&CA/EGCG/IG3/BCP (final concentration: 50 μM for CA/EGCG, 2.0 μM for IG3/BCP), respectively, and incubated at 37 °C for 1.0 h. Then, 1.0 mL of each mixture was transferred into one well of a 96-well plate that was pre-coated by Aβ42M/Aβ42O (final concentration: 0.2 μM, 100 μL per well) with or without CA/EGCG/IG3/BCP (final concentration: 50 μM for CA/EGCG, 2.0 μM for IG3/BCP) at 4 °C for 12 h, and incubated for additional 24 h at 37 °C, and then blocked with blocking solution [5% nonfat dry milk in TBS buffer (10 mM Tris-HCl, pH 7.5, 75 mM NaCl)] (150 μL per well) for 1 h at room temperature (21 °C). Next, 0.2 mL of supernatant was taken from each well, and the amount of bound BDNF was probed using anti-BDNF antibody at 37 °C for 1.0 h, followed by HRP-conjugated goat anti-mouse IgG H&L/HRP using tetramethylbenzidine (TMB). Fifteen minutes later, the absorbance of each well was measured at 450 nm. PBS (10 mM, pH 7.4) was used as a blank control for the Aβ42 samples. All experiments were performed in triplicate and repeated three times with different batches of Aβ42. The *K*_D_ value of BDNF was equal to the initial concentration (M) of free Aβ42 at which half of the BDNF was bound to the coated Aβ42M or Aβ42O.

### 3.6. Determination of the Effect of Aβ42M/Aβ42O with and Without BOs and CAs on the Cytological Efficacy of BDNF in Terms of Cell Survival by MTT Assay

SH-SY5Y and HT22 cells were plated into 96-well plates at a density of approximately 2.0 × 10^4^ cells per well in 100 µL of DMEM and incubated under standard conditions (37 °C, 5% CO_2_) for 4–6 h prior to conducting experiments. After replacing the culture medium with a fresh medium as outlined in Table 4 and culturing the target cells accordingly, cell viability for each group was determined using the conventional MTT (3-(4,5-dimethylthiazol-2-yl)-2,5-diphenyltetrazolium bromide) method. For anti-BDNF antibody-treated groups shown in Table 4, 0.5 h after the addition of the anti-BDNF antibody (final concentrations: 50 pg/mL) to the cells, BDNF (final concentration: 20.0 pg/mL), Aβ42M/Aβ42O (final concentrations 20 nM), CA/EGCG (final concentrations 0–100 μM), IG3/BCP (final concentrations: 0–2.0 μM), or the corresponding mixtures, which were premixed for 0.5 h at 4 °C, were added. The cells were then incubated under standard conditions (37 °C, 5% CO_2_) for 2 h. Untreated cells were used as the blank control (Ctrl0). The cultures were then washed with PBS (10 mM, pH 7.4) and cell viability in each well was assessed using conventional MTT (3-(4,5-dimethylthiazol-2-yl)-2,5-diphenyltetrazolium bromide) assay, as previously described [22]. All experiments were performed in triplicate and repeated across six independent cell batches.

### 3.7. Molecular Docking

The crystal structures of Aβ42 monomer (PDB ID: 1Z0Q), BDNF (PDB ID: 1B8M), and TrkB-neurotrophin-4 (NT4) (PDB ID: 1HCF) were downloaded from Protein Data Bank (http://www.rcsb.org) for molecular docking and comparison in this study. The 3D structure of Aβ42 trimer was built from crystal structures of the Aβ42 monomer using the program AutoDock 4.2.6 (https://autodock.scripps.edu/download-autodock4/) and compared to the model of Aβ trimer (PDB: 5HOX), and then, the Aβ42 trimer’s 3D structure was optimized through energy minimization and molecular dynamics simulation, as described previously [8]. The molecular dockings of BDNF to Aβ42 monomer/trimer and to TrkB were performed as described previously [3]. The integrity of all docking models was visually examined using Discovery Studio (DS) Visualizer 3.1 and, finally, all docking images were obtained.

### 3.8. Immunofluorescence (IF) Microscopy

Glass slides were placed in a 48-well plate and cells were seeded at a density of approximately 2.0 × 10^4^ per well in 200 µL of DMEM, then incubated under standard conditions (37 °C, 5% CO_2_) for 4–6 h. The culture medium was aspirated and fresh DMEM containing BDNF (final concentration: 1.0 nM) or its mixtures with Aβ42M/Aβ42O (final concentration: 0.2 μM) and/or CA/EGCG/IG3/BCP (final concentration: 100 μM for CA/EGCG and 2.0 μM for IG3/BCP) was added to each well. Cells were further cultured under identical atmospheric conditions for 48 h. Finally, the colocalization of BDNF with its receptor TrkB or p75 was evaluated using double-labeling immunofluorescence (IF) staining and corresponding quantitative analysis, as described previously [8]. BDNF was probed with a BF488-conjugated anti-BDNF antibody (green), while receptors TrkB and p75 were probed with AF594-conjugated anti-TrkB antibody (red) and AF647-conjugated anti-p75 antibody (red), respectively. The cellular nuclei were counterstained with DAPI (4’,6-diamidino-2-phenylindole) for 10 min. Fluorescence imaging was performed using a laser scanning confocal microscope (Zeiss LSM710, Oberkochen, Germany). Each experiment was performed in at least triplicate and repeated across six independent cell batches.

To quantitatively assess the colocalization of BDNF with the membrane receptors TrkB/p75, dual-channel confocal stacks were acquired and analyzed using Manders’ colocalization methodology. Specifically, Manders’ overlap coefficient (MOC) and colocalization coefficients (M1 and M2) were calculated as described previously [8]. The MOC metric (scale 0–1.0) of the merged image quantifies the spatial overlap between BDNF (green) and TrkB/p75 (red), while Manders’ colocalization coefficients (M1 and M2) represent the proportion of colocalized pixels for each color relative to its total pixels. All data were obtained from three biological replicates (*n* = 3).

### 3.9. Co-Immunoprecipitation (Co-IP) Assay

The extent of BDNF binding to its receptors in the presence or absence of Aβ42M/Aβ42O with or without BOs/CAs was quantitatively analyzed using a conventional Co-IP assay with protein A&G beads and anti-BDNF, anti-TrkB, and anti-p75 antibodies, following the manufacturers’ instructions and our prior optimization. Briefly, cells were plated at a density of approximately 5.0 × 10^5^ per well in 1.5 mL of DMEM and incubated under standard conditions (37 °C, 5% CO_2_) for 4–6 h. The culture medium was then aspirated and fresh DMEM containing BDNF (final concentration: 1.0 nM) or its mixture with Aβ42M/Aβ42O (final concentration: 0.2 μM) and/or CA/EGCG/IG3/BCP (final concentration: 100 μM for CA/EGCG and 2.0 μM for IG3/BCP) was added to each well. Cells not treated with the above agents were used as the control cells. Following a 48 h incubation under standard conditions (37 °C, 5% CO_2_), the cell culture plates were transferred to a cold room (4 °C). The culture medium was aspirated and the cells were washed twice with 1.0 mL of ice-cold PBS (10 mM, pH 7.4). Then, 150 μL of ice-cold lysis buffer containing protease and phosphatase inhibitors was added to each well. The cells were gently pipetted several times to ensure full contact with the lysis buffer, followed by a 10 min incubation on ice (to ensure proper lysis and solubilization of membrane proteins and lipids while maintaining structural integrity). Each cell lysate was transferred to an ice-cold 1.5 mL tube and centrifuged at 13,680 g for 10 min (4 °C). Finally, the cell lysis supernatants were collected and used as whole-cell lysate (WCL) samples for subsequent Co-IP assays.

For all Co-IP assays described in Table 5, the immunoprecipitation procedure was performed as follows: 1.0–2.0 μL of anti-TrkB/p75 antibody (equivalent to 1.0 μg of antibody) or a control IgG (1.0 μL) was incubated with 100 μL WCL in an ice-cold 1.5 mL tube with continuous rotation at 4 °C for 12 h. Subsequently, Protein-A&G beads obtained from 50 μL of protein-A&G slurry and pre-washed twice with ice-cold lysis buffer were added to the antigen–antibody complexes and incubated with rotation at 4 °C for an additional 12 h. Following immunoprecipitation, the samples were briefly centrifugated (10× *g*, 1 min, 4 °C) and the supernatants and pelleted beads were collected separately. Finally, the original WCL, the supernatants from Co-IP (SFC), and Co-IP samples (all from equal amounts of cell lysates) were resolved on either 8% or 10% SDS-PAGE gels. The receptor-bound BDNF in these samples was then detected and visualized by conventional Western blotting using anti-BDNF antibody. All immunoreactive bands were developed using HRP-conjugated secondary antibodies and visualized by enhanced chemiluminescence (ECL) detection. Experimental samples were normalized to the corresponding control samples (WCL, SFC, and Co-IP) obtained from control cells.

The grayscale intensity was quantitatively analyzed using ImageJ software (Version: 1.8.0) to quantify BDNF immunoblot bands. The percentage of BDNF abundance in each WCL, SFC, and Co-IP sample was calculated by dividing the mean intensity of the target band (A) by that of the corresponding control band (A_0_) (from the control group), then multiplying by 100% [(A/A_0_) × 100]. All experiments were performed in triplicate and repeated across three distinct cell batches.

### 3.10. Statistical Analysis

All data in this study were obtained from at least three different batch experiments, each with three parallel samples, and represent the mean ± standard error of the mean (SEM). The cells in each experimental group were measured in random order by two independent investigators. During image capture and analysis, the investigators were blinded to Aβ42 species and experimental group. Normality test and homogeneity of variance test were performed using the SPSS statistical analysis software (SPSS 28.0). For the data-met normality of distribution and homogeneity of variance, between-group comparisons were performed using Student’s *t*-test analysis, and *p* values smaller than 0.05 were considered to be statistically significant.

## 4. Discussion

Although numerous in vitro and in vivo studies have shown that the massive loss of cognitive neurons in AD is closely related to Aβ42 aggregates, the role of the extracellular Aβ42 aggregates, particularly Aβ42O, for NTs, especially BDNF, remains unknown. BDNF is the major neurotrophin in the CNS and is required for neuronal survival and growth, synaptic plasticity, and neurite outgrowth [31,32]. By binding to and activating its specific receptors, TrkB and p75, BDNF exerts powerful biological effects that maintain normal neuronal function and promote the repair of injured neuronal cells [13,33]. Numerous in vitro and in vivo studies have demonstrated that the activation or upregulation of the BDNF signaling pathway promotes neurogenesis and synaptogenesis, prevents cell death during degenerative processes, and improves learning and cognitive abilities in AD mice [34,35]. In contrast, insufficient or reduced BDNF leads to synaptic dysfunction or loss, which accelerates neuronal death and apoptosis [36,37] and has been implicated in the pathology of several neurodegenerative diseases as well as in their physiological symptoms [38]. AD is a neuropathy caused by the lack of neurotrophic factors [39,40,41]. Although the neurotrophic effects of Aβ42M and the neurotoxic effects of Aβ42 aggregates on neuronal cell survival are well-documented, the precise relationship between Aβ42M (or its aggregates) and BDNF remains poorly understood.

BOs and CAs have been shown to increase the beneficial effects of Aβ42M and decrease the deleterious effects of Aβ42O both in vitro and in vivo [15,16,17,18]. This study examined the interactions between Aβ42M/Aβ42 aggregates (especially Aβ42O) and BDNF and determined the effects of Aβ42M and Aβ42O on the BDNF receptor-binding activity and effectiveness in supporting cell survival and the roles of BOs and CAs in these processes. It was found that the affinity of BDNF for both Aβ42M and Aβ42O was higher than that of BOs for Aβ42M and Aβ42O [15], but both were lower than that of BDNF for its receptors [29], as indicated by the dissociation constants in Table 1. Additionally, the affinity of BDNF for Aβ42O was marginally higher than that for Aβ42M (Table 1). BDNF is a basic protein with an isoelectric point (pI) of approximately 9.6 and consists of 119 amino acid residues, including 12 acidic (Asp + Glu) and 22 basic (Arg + Lys) residues. As a result, numerous polar groups or motifs are present on the surface of the BDNF molecule. Under physiological conditions, BDNF readily binds to the amphiphilic N-terminal fragment of amphipathic Aβ42 molecules (pI ≈ 5.4) [1]. However, the hydrophilic N-terminal fragment of the Aβ42 peptide chain is rarely exposed on the outer surface of the larger Aβ42 aggregate particles [1]. Thus, BDNF binds easily to Aβ42M and smaller Aβ42O particles, but hardly binds to larger Aβ42P and Aβ42F units (Figure 1). Furthermore, the large size of the Aβ42P/Aβ42F units reduces the probability of close spatial proximity to BDNF, resulting in suboptimal binding kinetics with a binding rate of <5% and low affinity. Since the accessible surface area between BDNF and Aβ42O was larger than that between BDNF and Aβ42M (Figure 3), the binding affinity between BDNF and Aβ42O was slightly higher than that between BDNF and Aβ42M (Table 1), and the stability of the BDNF-Aβ42O complex was greater than that of the BDNF-Aβ42M complex (Table S1). These differential binding properties may mechanistically explain the paradoxical functional outcomes of Aβ42M versus Aβ42O in modulating BDNF-supported neuronal survival.

Since very little BDNF is produced in the CNS and cell culture systems [25,26,27], even minor changes in the extracellular BDNF levels can impact cell survival. The neurotrophic effect of Aβ42M may primarily result from its ability to synergistically enhance the efficacy of BDNF because this study showed that Aβ42M increased neuronal cell survival via BDNF (Figure 2) and promoted BDNF binding to its receptors (Table 2 and Table 3). These findings suggest that the physiological significance of the specific and reversible BDNF-Aβ42M binding may stem from the Aβ42M-mediated surface recruitment of BDNF and/or the synergistic potentiation of BDNF-receptor engagement. In contrast, Aβ42O exhibits neurotoxic properties through high-affinity, less reversible BDNF-Aβ42O binding, which competitively blocks BDNF-receptor interactions (Table 2 and Table 3). Such pathological sequestration depletes bioactive BDNF in the extracellular matrix (Figure 2), thereby reducing BDNF-supported neuronal viability. Thus, BDNF binding by Aβ42O is equivalent to Aβ42O-induced BDNF inactivation or consumption, which may represent the primary (though not exclusive) mechanism of Aβ42O’s neurotoxicity. Therefore, the quantity of functional extracellular BDNF, rather than the total quantity of BDNF present, determines the cytological efficacy of BDNF.

Notably, neurotoxic Aβ42O was also consumed through the formation of BDNF-Aβ42O complexes; that is, Aβ42O and BDNF neutralized or counteracted each other’s effects on neuronal cell viability when Aβ42O and BDNF specifically bound together. The level of either Aβ42O or BDNF appeared to influence the extent to which the other was consumed or inactivated. Consequently, BDNF would be progressively consumed as Aβ42O levels in the brain gradually increase, leading to a gradual decline in neuronal cell survival. As others have reported [26,42,43], upregulating BDNF expression or secretion could potentially mitigate the neurotoxicity of extracellular Aβ42O. In summary, a significant portion of the damage and death of neurons induced by Aβ42O or other Aβ42 aggregates may result from the depletion or inactivation of BDNF by these aggregates, whereas a significant portion of the improved neuronal survival caused by Aβ42M may be due to the enhanced potency of BDNF by Aβ42M.

This study also found that by boosting the beneficial synergistic effect of Aβ42M on BDNF binding to its receptors and inhibiting Aβ42O from consuming extracellular BDNF (Table 2 and Table 3), BOs and CAs were able to increase the amount or proportion of BDNF bound to its receptors in the presence of Aβ42M or Aβ42O. This should be the result of their competitive displacement of BDNF for binding to the Aβ42M and Aβ42O, despite the lower-affinity BOs and Aβ42M/Aβ42O compared to BDNF (Table 1) [15]. The binding of BOs and CAs to Aβ42M/Aβ42O promoted the synergistic effect of Aβ42M on BDNF, stabilized the bioactive conformation of Aβ42M, and inactivated Aβ42O. These findings demonstrate that reduced neuronal viability was likely attributable to insufficient effective BDNF caused by Aβ42O. Therefore, the previously reported neuroprotective effects of BOs and CAs may be attributed to their indirect beneficial effect on BDNF activity, altering the interaction between Aβ42M/Aβ42O and the BDNF.

**Figure 6:**
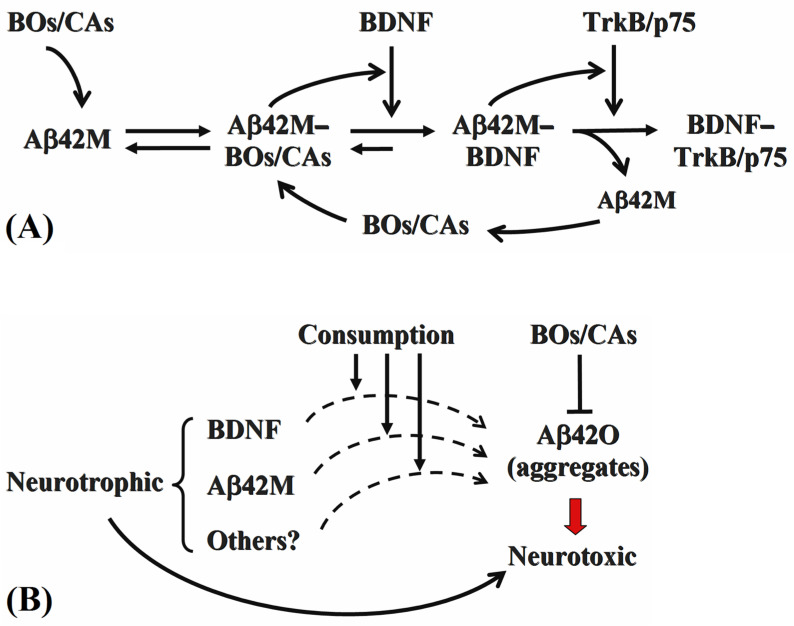
Schematic illustration of the different effects of Aβ42M (**A**) and Aβ42O (**B**) on BDNF and the modulatory roles of BOs and CAs therein.

In addition to being present in the extracellular matrix, BOs are more likely to be located on the outer surface of the cytoplasmic membrane as a component of membrane glycolipids, glycoproteins, or receptors [15]. Therefore, BOs were likely to interact with Aβ42M or Aβ42O close to or on the outer surface of the cytoplasmic membrane. This implies that the synergistic effect of Aβ42M on BDNF binding to its receptor and the consumption of BDNF by Aβ42O might both occur in a membrane-proximal manner. Accordingly, Aβ42M may elevate the localized concentration of bioactive BDNF within the pericellular microenvironment, whereas Aβ42O depletes it. Moreover, BOs and CAs, through their interaction with both Aβ42M and Aβ42O, simultaneously enhance the Aβ42M-mediated potentiation of BDNF-receptor engagement and attenuate Aβ42O-mediated BDNF consumption, thus exerting neuroprotective effects. Therefore, although BOs/CAs improve or enhance the potency of BDNF in both Aβ42M and Aβ42O systems, the underlying mechanisms may differ. Figure 6 summarizes the findings of this study.

Aβ42 aggregates, especially Aβ42O, are considered the neurotoxic and pathogenic form in AD. Given the lack of successful therapeutic approaches for AD, targeting the elimination of Aβ42O neurotoxicity at an early stage holds the potential for the development of new, effective AD treatments. However, the findings of this study also imply that while Aβ42 aggregates are the characteristic pathological features of AD in the brain, making these aggregates, plaques, and downstream dysfunctions the main focus, we should also pay attention to their initial beginning, i.e., the Aβ42M. The findings of this study indicate that an abnormal decrease in endogenous BOs-like molecular chaperones would inevitably increase the likelihood of Aβ42M aggregation and a consequent BDNF deficiency. Therefore, further research should be completed on using nanotechnology to create nanomaterials for exogenous BOs or CAs to improve their bioabsorption or bioavailability.

This study has some limitations. First, this study focused solely on BDNF, not other neurotrophins. Although BDNF is the main neurotrophin in the CNS, other neurotrophins, such as NGF, NT3, and NT4/5, also exist. They possess similar but not identical conformational and functional properties to those of BDNF. Therefore, Aβ42 or its aggregates may interact with them in similar yet distinct ways. The scope for future studies can be expanded to include investigating the effects of other neurotrophins, such as NGF and NT3, on neuronal survival in AD. Second, this study investigated the role of CA/EGCG/IG3/BCP-represented BOs and CAs in affecting BDNF’s cytological functions through Aβ42M and Aβ42O. These findings offer encouragement for preventing and potentially treating AD. However, the wide variety of polyhydroxy or polyphenolic chemicals in plants implies a diversity of physiological functions. The role of other polyhydroxy or polyphenolic chemicals requires further exploration.

In conclusion, this study found that (1) compared to BOs and CAs, BDNF had a higher binding specificity and affinity for Aβ42M and Aβ42O; (2) the neurotrophic effect of Aβ42M was closely linked to Aβ42M’s ability to preserve the amount of effective BDNF in the extracellular matrix, close to the cytoplasmic membrane, and/or to enhancing the potency of the BDNF to bind to TrkB and p75 receptors, whereas the neurotoxicity of Aβ42O was directly related to Aβ42O’s ability to inactivate or consume BDNF in the extracellular matrix, close to the cytoplasmic membrane, and/ or reducing the efficiency of BDNF to bind to TrkB and p75 receptors; (3) BDNF and Aβ42O counteracted each other’s effects on neuronal cell viability when they bound together; and (4) BOs and CAs, mainly CA, EGCG, IG3, and BCP, enhanced or increased the synergistic effect of Aβ42M on BDNF and inhibited or decreased the predatory or consuming effect of Aβ42O on BDNF. These findings provide important insights for developing BOs- and plant polyphenol-based neuroprotective medications and their potential clinical applications in AD treatment. Clinically, these findings support novel neuroprotective strategies targeting BDNF modulation. Policy implications include prioritizing BDNF-based interventions. Future research should explore broader neurotrophin interactions. Further research on the effects of Aβ42M and Aβ42O on BDNF and other neurotrophins will define the critical step in the pathogenesis of AD.

## Figures and Tables

**Table 1:** *K*_D_ values (M) for BDNF.

Aβ42 Species	In the Presence of ^a^ CA (or EGCG), ^b^ IG3, or ^c^ BCP	In the Absence of CA/EGCG/IG3/BCP
Aβ42M	^a^ 4.91 ± 0.57 × 10^−7^	6.67 ± 0.26 × 10^−7^
	^b^ 3.89 ± 0.43 × 10^−7^	
	^c^ 6.17 ± 0.13 × 10^−7^	
Aβ42O	^a^ 3.36 ± 0.27 × 10^−6^	2.88 ± 0.33 × 10^−7^
	^b^ 2.19 ± 0.12 × 10^−6^	
	^c^ 2.34 ± 0.28 × 10^−5^	

Aβ42M, Amyloid-β monomers; Aβ42O, oligomers; CA, catechins; EGCG, epigallocatechin gallate; IG3, isomaltotriose; BCP, bianntennary N-linked core pentasaccharide; M, molar concentration; ^a^, CA or EGCG group; ^b^, IG3; ^c^, BCP group.

**Table 2:** Parameters ^a^ MOC, ^b^ M1, and ^c^ M2.

No.	Group	MOC	M1	M2	Receptor
1	Control	0.622 ± 0.013	0.587 ± 0.004	0.611 ± 0.001	TrkB
2	Control	0.614 ± 0.007	0.556 ± 0.006	0.200 ± 0.003	p75
3	BDNF (or with BOs/CAs)	0.778 ± 0.009	0.653 ± 0.002	0.638 ± 0.004	TrkB
4	BDNF (or with BOs/CAs)	0.654 ± 0.011	0.565 ± 0.004	0.208 ± 0.007	p75
5	AβM+BDNF+CA/EGCG	0.714 ± 0.015	0.586 ± 0.006	0.668 ± 0.004	TrkB
6	AβM+BDNF+BCP	0.823 ± 0.009	0.592 ± 0.003	0.639 ± 0.002	TrkB
7	AβM+BDNF+IG3	0.871 ± 0.012	0.597 ± 0.001	0.702 ± 0.005	TrkB
8	AβM+BDNF+HT6	0.731 ± 0.005	0.593 ± 0.004	0.670 ± 0.003	TrkB
9	AβM+BDNF	0.721 ± 0.006	0.551 ± 0.007	0.650 ± 0.005	TrkB
10	AβM+BDNF+CA/EGCG	0.875 ± 0.010	0.598 ± 0.006	0.316 ± 0.002	p75
11	AβM+BDNF+BCP	0.726 ± 0.007	0.583 ± 0.002	0.295 ± 0.003	p75
12	AβM+BDNF+IG3	0.746 ± 0.012	0.566 ± 0.005	0.309 ± 0.002	p75
13	AβM+BDNF+HT6	0.726 ± 0.012	0.534 ± 0.005	0.328 ± 0.003	p75
14	AβM+BDNF	0.710 ± 0.005	0.536 ± 0.003	0.271 ± 0.002	p75
15	AβO+BDNF+ CA/EGCG	0.676 ± 0.011	0.517 ± 0.005	0.523/0.581 ± 0.004	TrkB
16	AβO+BDNF+BCP	0.619 ± 0.012	0.623 ± 0.005	0.686 ± 0.004	TrkB
17	AβO+BDNF+IG3	0.623 ± 0.011	0.557 ± 0.004	0.656 ± 0.003	TrkB
18	AβO+BDNF+HT6	0.769 ± 0.015	0.550 ± 0.005	0.673 ± 0.004	TrkB
19	AβO+BDNF	0.617 ± 0.008	0.410 ± 0.003	0.572 ± 0.001	TrkB
20	AβO+BDNF+CA/EGCG	0.661 ± 0.009	0.524 ± 0.002	0.300 ± 0.002	p75
21	AβO+BDNF+BCP	0.672 ± 0.006	0.533 ± 0.006	0.302 ± 0.003	p75
22	AβO+BDNF+IG3	0.712 ± 0.015	0.544 ± 0.004	0.327 ± 0.005	p75
23	AβO+BDNF+HT6	0.817 ± 0.011	0.561 ± 0.007	0.311 ± 0.003	p75
24	AβO+BDNF	0.615 ± 0.007	0.485 ± 0.004	0.163 ± 0.002	p75

^a^ MOCs (Manders’ overlap coefficients) were calculated based on the merged whole images. The MOC value from 0 to 0.6 indicates absence of colocalization, and that from 0.6 to 1.0 indicates colocalization. ^b^ M1 and ^c^ M2 (Manders’ colocalization coefficients) were calculated by analyzing the corresponding fluorescence signals corresponding to the merged images. M1: fraction of TrkB or p75 overlapping BDNF; M2: fraction of BDNF overlapping TrkB or p75. All data were the mean ± SEM. BOs/CAs: branched oligosaccharides/catechins.

**Table 3:** Mean percentage of BDNF bound to TrkB and p75 by quantitative analysis of BDNF immunoblots in Co-IP.

Group	Bait	BDNF	Aβ42M	Aβ42O	CA/ EGCG	IG3/ BCP	WCL	Co-IP	SFC
a		−	−	−	−	−	100	44.07 ± 0.79	10.87 ± 1.09
d	TrkB	+	−	−	−	−	243.90 ± 1.31	85.03 ± 0.58	63.22 ± 0.64
f		+	+	−	−	−	248.71 ± 1.51	88.33 ± 0.96	51.63 ± 0.74
g		+	+	−	+	−	242.51 ± 0.89	89.27 ± 0.65	47.72 ± 0.88
h		+	+	−	−	+	245.97 ± 1.37	92.01 ± 0.84	46.39 ± 0.73
l		+	−	+	−	−	210.06 ± 1.08	58.12 ± 0.74	56.13 ± 0.61
m		+	−	+	+	−	221.36 ± 0.95	72.18 ± 0.87	45.60 ± 0.72
n		+	−	+	−	+	212.38 ± 1.35	73.44 ± 0.63	49.61 ± 0.76
b		−	−	−	−	−	100	31.95 ± 1.04	12.57 ± 0.42
e	P75	+	−	−	−	−	243.90 ± 1.31	70.00 ± 0.69	22.81 ± 0.73
i		+	+	−	−	−	248.29 ± 1.31	71.88 ± 0.67	39.11 ± 0.78
j		+	+	−	+	−	242.65 ± 1.26	73.40 ± 0.78	38.07 ± 0.91
k		+	+	−	−	+	246.20 ± 1.53	83.23 ± 0.68	37.58 ± 0.73
o		+	−	+	−	−	208.18 ± 1.38	64.28 ± 0.63	43.90 ± 0.54
p		+	−	+	+	−	230.02 ± 1.32	62.07 ± 0.76	47.02 ± 0.62
q		+	−	+	−	+	231.36 ± 0.62	61.68 ± 0.98	41.85 ± 0.81

The grayscale intensity of the corresponding BDNF bands from the control WCL samples was considered as 100%. The symbols indicating groups listed in Table 3 correspond to those in Figure 5. WCL: whole-cell lysate samples. SFC: supernatants from Co-IP. Co-IP: Co-IP beads. Data for BDNF+Aβ42M and BDNF+Aβ42O+IG3 groups in Table 3 (n and i) were normalized with their multiplicative scores shown in Figure 5 (n and i). −, absent; +, present. All data were the mean ± SEM.

**Table 4:** Experimental groups for MTT assay.

Group	Incubated Duration (h)	Anti-BDNF Antibody	BDNF	Aβ42M/ Aβ42O	CA/EGCG/ IG3/BCP
Ctrl0	2	−	−	−	−
①	2	+	−	−	−
②		−	+	−	−
③/④		−	−	+	−
⑤	0.5 + 1.5	+	+	−	−
⑥		+	−	−	+
⑦/⑧		+	−	+	−
⑨/⑩	2	−	+	+	−
⑪/⑫	0.5 + 1.5	−	−	+ premixture	
⑬/⑭		+	+	+	−
⑮/⑯		+	−	+ premixture	
⑰/⑱		+	+	+ premixture	
⑲/⑳		−	+	+ premixture	

Note: −, absent; +, present.

**Table 5:** Experimental groups for Co-IP assay.

Group	Bait	Prey	BDNF	Aβ42M	Aβ42O	CA/EGCG/ IG3/BCP
a	TrkB	BDNF	−	−	−	−
d			+	−	−	−
f			+	+	−	−
g/h			+	+	−	+
l			+	−	+	−
m/n			+	−	+	+
b	p75		−	−	−	−
e			+	−	−	−
i			+	+	−	−
j/k			+	+	−	+
o			+	−	+	−
p/q			+	−	+	+

Note: −, absent; +, present.

## Data Availability

The datasets and materials used and/or analyzed during the current study are available from the corresponding author upon reasonable request.

## Supplementary Materials

The following supporting information can be downloaded at: https://www.mdpi.com/article/10.3390/ijms26104501/s1.

## Abbreviations

Aβ42	amyloid-β protein 1–42
AD	Alzheimer disease
CAs	catechins
EGCG	epigallocatechin gallate
BOs	branched oligosaccharides
IG3	isomaltotriose
BCP	bianntennary N-linked core pentasaccharide
BDNF	brain-derived neurotrophic factor
NT	neurotrophin
NGF	nerve growth factor
TrkB	tyrosine kinase receptor B
ELISA	enzyme-linked immunosorbent assay
BBB	blood–brain barrier
DMEM	Dulbecco’s modified eagle’s medium
FBS	fetal bovine serum
HSA	human serum albumin
