# Supplementary material for: Neurotrophic and Neurotoxic Effects of Aβ42 and Its Oligomers on Neuronal Survival: Revealed by Their Opposite Influence on the Potency of Extracellular BDNF
Source: Int J Mol Sci. 2025 May 8;26(10):4501. doi: 10.3390/ijms26104501 (PMC12111036; doi:10.3390/ijms26104501)
Supplement: Supplementary file 1 [file ijms-26-04501-s001.zip › ijms-3565364-supplementary.pdf]

Supplementary materials

Neurotrophic and neurotoxic effects of Aβ42 and its oligomers on neuronal survival: revealed by their opposite influence on the potency of extracellular BDNF

He Li, Changxin Zheng, Kai Wen, Tianyu Zhang, Yingjiu Zhang*

Key Laboratory for Molecular Enzymology and Engineering of the Ministry of Education, School of Life Sciences, Jilin University, Changchun 130012, China

*Correspondence: yingjiu@jlu.edu.cn

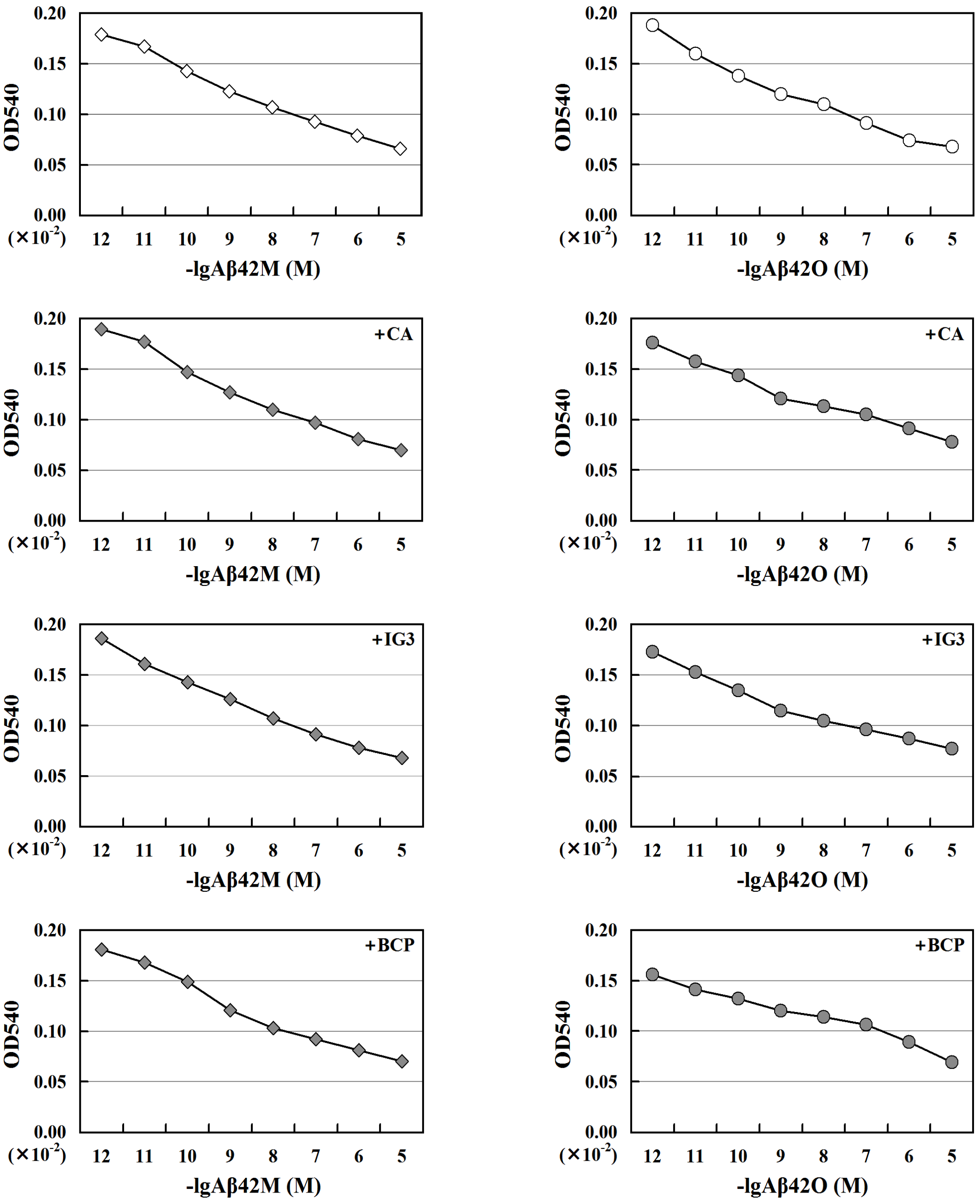

Fig. S1 Analysis of the binding affinity of BDNF to Aβ42M and Aβ42O, respectively, by Sandwich ELISA.
(M): Molar concentration ofAβ42.

Table S1 Energies by molecular docking of BDNF and Aβ42 monomer/trimer.

Aβ42 Species	Energy (kcal/mol)		
	E_{elec}	E_{vdw}	E_{inter}
Aβ42 Monomer	-372.0	-41.0	-413.0
Aβ42 Trimer	-386.8	-41.7	-428.5

The interaction energy (E_{inter}) includes the electrostatic energy (E_{elec}) and van der Waals interaction (E_{vdw}). The value of the interaction energy is inversely proportional to the degree of interaction.
